# Implementation and use of cloud-based electronic lab notebook in a bioprocess engineering teaching laboratory

**DOI:** 10.1186/s13036-017-0083-2

**Published:** 2017-11-24

**Authors:** Erin M. Riley, Holly Z. Hattaway, P. Arthur Felse

**Affiliations:** 1 Master of Biotechnology Program, Northwestern University, Evanston, IL 60208 USA; 2 Department of Chemical and Biological Engineering, Northwestern University, 2145 Sheridan Road, TECH E136, Evanston, IL 60208 USA; 3 Present address: Catalent Pharmaceuticals, 726 Heartland Trail, Madison, WI 53717 USA

**Keywords:** Electronic lab notebook, Good documentation practice, Data integrity, Experiment workflow, Pedagogy

## Abstract

**Background:**

Electronic lab notebooks (ELNs) are better equipped than paper lab notebooks (PLNs) to handle present-day life science and engineering experiments that generate large data sets and require high levels of data integrity. But limited training and a lack of workforce with ELN knowledge have restricted the use of ELN in academic and industry research laboratories which still rely on cumbersome PLNs for recordkeeping. We used LabArchives, a cloud-based ELN in our bioprocess engineering lab course to train students in electronic record keeping, good documentation practices (GDPs), and data integrity.

**Results:**

Implementation of ELN in the bioprocess engineering lab course, an analysis of user experiences, and our development actions to improve ELN training are presented here. ELN improved pedagogy and learning outcomes of the lab course through stream lined workflow, quick data recording and archiving, and enhanced data sharing and collaboration. It also enabled superior data integrity, simplified information exchange, and allowed real-time and remote monitoring of experiments. Several attributes related to positive user experiences of ELN improved between the two subsequent years in which ELN was offered. Student responses also indicate that ELN is better than PLN for compliance.

**Conclusions:**

We demonstrated that ELN can be successfully implemented in a lab course with significant benefits to pedagogy, GDP training, and data integrity. The methods and processes presented here for ELN implementation can be adapted to many types of laboratory experiments.

## Background

Data recording and reporting is of highest importance in all types of research. Data that is not recorded or recorded incorrectly is summarily invalid. Academic teaching laboratory courses have emphasized the importance of accurate record keeping and extensively trained students in good documentation practices (GDPs) based on paper lab notebooks (PLNs). Though the use of PLNs has been perfected over several decades, the large data sets generated by many contemporary life science experiments are better managed through electronic laboratory notebooks (ELNs). But the academic community has been generally slow in moving towards the use of electronic laboratory notebooks [1, 2]. Lack of resources, unstandardized regulations, data security concerns, and low activation energy for changes contribute to poor adoption of ELN in the academia [3]. As a result, only about 5% of academic labs use ELN [4]. Agencies such as the National Institutes of Health (NIH) routinely emphasis the importance of data sharing and reproducibility. A report from the NIH concluded that the main reason for non-reproducibility of research data is the lack of good documentation methods rather than scientific misconduct [5]. ELNs can facilitate data sharing and simplify good documentation practices, and subsequently improve reliability of scientific data better than PLNs [6]. Also, ELNs can simplify recording and archiving of large data sets such as those generated in -omics research and in core laboratories [7].

Academic laboratories are beginning to adopt ELNs encouraged by the recent availability of several open source, cloud-based ELN software for life science research [8]. Many vendors have launched no- or low-cost versions of ELN software for academic use [9]. Machina and Wild [10] in their review article categorize the pros, cons, difficulties, and success factors in implementing ELNs in academia. Rubacha et al., classified 35 commercial ELNs in the market and developed guidelines to select the right ELN based on user requirements [11]. A recent study identified cost and incompatibility across operating systems as the key barriers for adoption of ELNs in the academia, and provided a framework to build future ELNs based on user feedback results [12]. Some academic laboratories have developed surrogate ELNs by adapting software that were originally not intended for data recording. Examples include the use of Evernote, Googledocs, Microsoft OneNote, and web blogs as ELNs [13–16]. In addition, the following studies on inclusion of ELN in teaching laboratories have been reported in the literature: An ELN based on the Sakai software was used in an inquiry-based biochemistry teaching laboratory course [17], and the Pebblepad ePortfolio system was used as ELN in a biochemistry and molecular biology lab course [18]. Weibel described the use of Googledocs for a paperless undergraduate physical chemistry teaching laboratory [19].

**Fig. 1:**
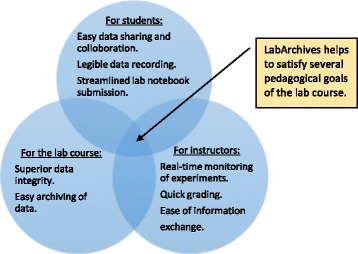
Pedagogical goals accomplished through ELN

In this paper we present the implementation and use of LabArchives, a cloud-based ELN software in our bioprocess engineering laboratory course. The multitude of pedagogical objectives accomplished through the use of LabArchives ELN are summarized in Fig. 1. For students LabArchives facilitated legible and quick data recording, improved data sharing and collaboration, and streamlined the lab notebook submission process. For instructors, it facilitated real-time monitoring of experiment workflow, ease of grading and feedback, and simplified information sharing with students. For the lab course, it enabled superior data integrity and quality through reliable audit trails, and efficient archiving of data. We also present an analysis of students’ responses on the use of ELN, our development actions to improve student experience, and propose future directions.

### Rationale for using ELN in a teaching laboratory

PLNs have been used by both academia and the industry even since data collection began [20]. PLNs are still widely used but they cannot accommodate the large sets of data generated by today’s life science experiments [21]. Legibility, data integrity, security, and archiving of PLNs are a huge logistical and financial burden. Inefficiencies inherent in using PLNs costs the drug industry about $1 billion annually by way of lost data sharing opportunities and redundancy in data generation [22]. ELNs can effectively address several disadvantages associated with PLNs. ELNs facilitate better workflow, quick data retrieval, remote accessibility of data, and enables superior data integrity. A recent article in *Science Careers* identified knowledge in using ELNs as one of the key tools for successful careers in the science and technology industry [23]. We included ELN training in the bioprocess engineering lab course to better prepare students for careers in the biotechnology industry, to streamline workflow in the teaching lab, to facilitate data sharing and collaboration among student teams, and to create a culture of ELN use for the future workforce.

## Methods

### The bioprocess engineering lab course

The bioprocess engineering lab course is a graduate course offered to students in the Master of Biotechnology Program at Northwestern University. The lab course includes hands-on training in bench-scale upstream and downstream bioprocessing methods, good laboratory practice, design of experiments, quality and validation, team skills, and written communication skills. Experiments are done in three- or four-member student teams, and teams perform experiments on a rotating schedule. Students are expected to share data between teams and collaborate in data analysis. All experiments need data collection at multiple time points by different team members which should be recorded using a streamlined process. Experiments in this lab course generate extensive digital data (such as preparative chromatography and bioreactor experiments) which are exported as Excel or CSV files. Students in this lab course primarily have undergraduate degrees in biology, biotechnology, or chemical engineering. Students are distributed in teams based on their educational and cultural backgrounds, gender, and personality type (based on Meyers-Briggs personality type assessment).

### Implementation of ELN in the bioprocess engineering lab course

**Fig. 2:**
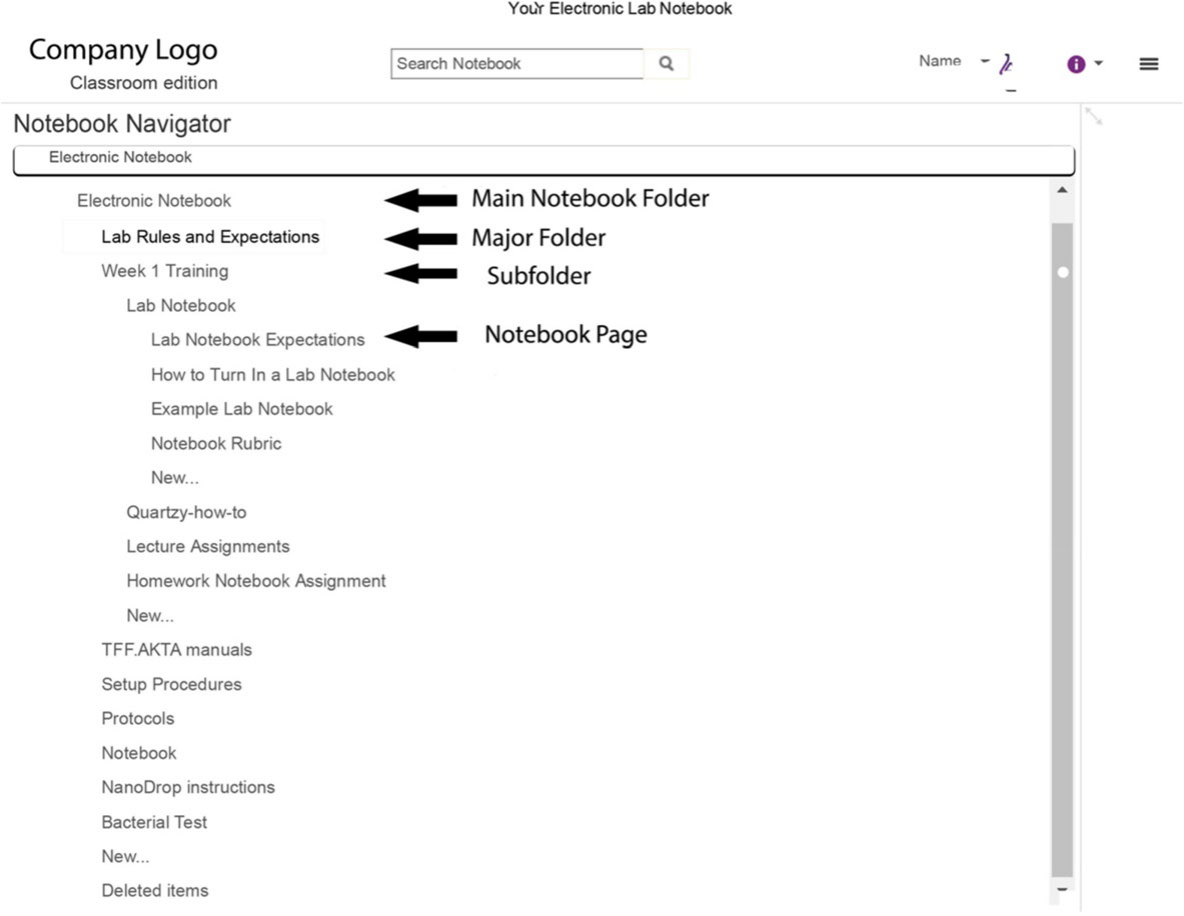
Screenshot of LabArchives’ document and folder system. All indented titles are contained within the folders above them

LabArchives subscriptions were purchased at about the same cost as PLN on a per student basis. Each student created an individual account and therefore we were able to track each entry with username and time stamp. The instructor populated the master ELN with folders and files with information on all experiments. Information included list of supplies and chemicals needed for the experiment, equipment operating manuals, calibration and preparation procedures, safety information on equipment, and safety data sheet documents. LabArchives was the single point source for information retrieval and data recording. All folder structures and information contained in the folders were then cloned into student notebooks as shown in Fig. 2. LabArchives allows for regular update of information provided by the instructor, so it was possible to communicate new information to students instantly as they became available.

Once students were placed in teams, each team chose a notebook leader in whose electronic notebook the data would be submitted for the entire term. The team notebook leader then shared their notebook and gave the team members the ability to view and edit the notebook as well as to turn in the assignments for grading. The assignments could be submitted by checking the assignment submission button at which point the notebook is unable to be further edited, and timestamps ensured that the notebooks were submitted on time. The team notebook leader was also able to share their notebook with students in other teams by giving them view-only privileges to certain pages. This guaranteed data integrity while allowing the teams to share their data with others for collaborative learning. Revisions made to LabArchives entries by students are tracked collectively for the team based on the team’s (notebook leader’s) user identification. But it was required for the student making the revisions to identify themselves and enter the reason for revision in the comments section. Thus all revisions were attributed to a particular student making the revision. Revisions made without identity were considered to be non-compliant and invalid. Attribution at the student level promotes strict compliance from all students, and makes it possible to trace the complete history of changes if necessary.

### ELN user experience evaluation

After the bioprocess engineering lab course offerings in 2015 and 2016, students were asked to complete an exit survey on the use ELN for course development proposes. SurveyMonkey was used to deploy the survey questions and collect feedback. The survey included queries related to ELN use, introductory lecture, and compliance, among others. Data related to student experiences in using ELN were extracted from this survey and analyzed. The lab course had enrollments of 38 and 33 students during 2015 and 2016 respectively. Student response counts for the survey were 32 (84.2% response rate) and 23 (70% response rate) during 2015 and 2016 respectively. All students who responded to the survey completed it in entirety.

Survey queries used in this study are shown in Table 1. Student experiences on various attributes important to ELN use were assessed using the Likert Scale (Query 1). Statistical significance of differences in student responses between 2015 and 2016 course offerings were evaluated using a two sample *t*-test assuming unequal variances. Time taken for the students to become comfortable with ELN was quantified as number of weeks (Query 2). Time taken for students to become comfortable with ELN use in a particular year was estimated using a weighted average composite score defined as,

$$ Composite score=\varSigma \left( time taken to become comfortable\kern0.5em \times \kern0.5em \% of students reporting this time\right) $$

**Table 1:** Queries used in user experience evaluation survey

Query 1: Please rate your experiences in the following attributes in using ELN					
Attribute	Score (pick one)				
	1 (poor)	2 (passable)	3 (neutral/adequate)	4 (very good)	5 (excellent)
Introductory lecture					
Submission					
Data sharing					
Accessibility					
Query 2: How many weeks did it take for you to become comfortable with ELN software (pick one)?					
1	2	3	4	5	Still not comfortable
Query 3: Which system makes it easier to comply with academic rules and expectations for the lab course (pick one)?					
Electronic is easier		Paper is easier		Both are same	
Query 4: Please rate your experiences in completing the following tasks in ELN compared to PLN					
Task	Score (pick one)				
	1 (ELN is much worse than PLN)	2 (ELN is worse than PLN)	3 (ELN and PLN are the same)	4 (ELN is better than PLN)	5 (ELN is much better than PLN)
Data entry					
Adding figures					
Data sharing					
Editing information					

Students reporting “still not comfortable” were assigned six weeks which is the minimum time it would take for them to become comfortable with ELN. Student perceptions on ELN vs. PLN for compliance with academic rules and expectations was queried using a multiple choice question (Query 3). Comparison of student experiences in completing documentation and data sharing tasks using ELN vs. PLN was assessed using the Likert Scale (Query 4).

## Results and discussion

### Experiment workflow using LabArchives

**Fig. 3:**
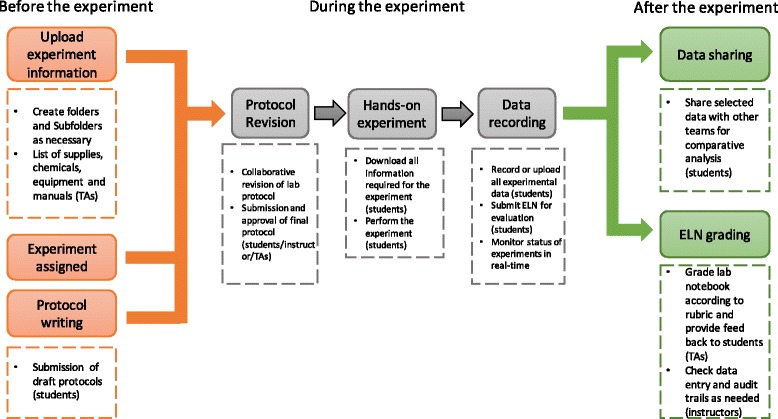
General workflow of the lab experiment showing the role of ELN. Tasks that were done through LabArchives are shown in boxes with broken lines

The general workflow and use of LabArchives in each step of an experiment is shown in Fig. 3. Each experiment had a set of tasks to be completed by different people before, during, and after the experiment. LabArchives was used to manage these tasks in a streamlined manner. LabArchives also provided a platform for interactive exercises such as protocol revision.

### Protocol writing

Students teams were assigned experimental goals ahead of time. They were required to write a draft experimental protocol and submit it on LabArchives before the start of experiment. A subfolder was created for protocols under each experiment folder. The first activity on the experiment day was protocol review by the instructor or the teaching assistant (TA). Students were typically asked about the concepts behind the experiment, rationale for the experimental steps, and an explanation of calculations and data analysis that they intend to do after data acquisition. Students and the instructors/TA collectively revised the experimental protocol on LabArchives. After the protocol was finalized, the instructor/TA approved it electronically. Since LabArchives maintains a record of changes made along with time stamp and user identity, it was easy to track all the changes that were made to the protocol. Students can see in real time how their protocol has evolved from an initial draft to the final form. Pedagogically, protocol writing is an exercise in translating theoretical concepts to an experimental procedure. The collective revision of protocol by students and instructor lends well to hands-on, inquiry-based learning [17]. Unlike PLNs, LabArchives allows for multiple revisions and the final version is clear and legible. The protocol review exercise takes about 30 min.

### Hands-on lab work and data recording

Students performed the experiment according to the revised protocol. Data was directly recorded in LabArchives by students. Machine acquired data was transferred from the lab equipment (such as chromatography data) through a storage device (such as flash drive) and manually uploaded to LabArchives by students. LabArchives does not have the functionality for real-time data entry from lab equipment. All information that students will need to do an experiment was ready for download from LabArchives. LabArchives provides a robust audit trail of changes made to data entry which allows the instructor to review data change history and access metadata. Instructors can view data entry in real time to monitor progress of the experiment. This allows the instructors to identify mistakes and intervene immediately rather than ponder what could be done differently after the experiment has been completed. LabArchives thus facilitates better engagement between the instructor and students through online contact in addition to in-person interactions.

### Data sharing and collaboration

Each experiment was done by two student teams during any given week, but the teams had different experimental conditions. Students were required to share data between teams for comparative data analysis. LabArchives allows teams to selectively share data with other teams. Students can now readily share large data sets without the need for photocopies or sending data sets and PLN pages as e-mail attachments. Since data sharing also leaves an audit trail, the instructor can check if data is being shared in a timely manner. Because students know the teams they need to share the data with, it is also possible to schedule data sharing ahead of time for the entire course.

### Data integrity using ELN

Data integrity is of supreme importance in scientific investigations and the validity of new scientific knowledge is dependent on the quality of data that is generated. Elements of data integrity are best defined in the Food and Drug Administration’s (FDA’s) guidance on data integrity requirements, which state that all scientific data should be Attributable, Legible (and long-lasting), Contemporaneously recorded, Original, and Accurate (or ALCOA) [24]. ELN permits full life-cycle data management from creation to archiving while incorporating all aspects of ALCOA. LabArchives uses exclusive login for each student to access their lab notebook. Instructors (as account administrators) have the ability to restrict access or terminate users as needed. Users can retrieve lost passwords through a secure password retrieval process using the e-mail address linked to their account. User login are recorded and archived, and can be retrieved by the instructor. Therefore, any entry made in LabArchives is attributable to the user making the entries. Since data is directly entered or uploaded in LabArchives, all data is legible unlike in PLNs which tend to have scratched out and overwritten data.

According to the FDA guidance, audit trail is defined as “secure, computer-generated, time-stamped electronic record that allows for reconstruction of the course of events relating to the creation, modification, or deletion of an electronic record” [25]. Any entry or upload in LabArchives will have an audit trail, i.e., user identity and time stamp for all entries, modifications, deletions, etc., will be recorded and available for audit by the instructor. Students were allowed to modify data, but modifications should be explained and justified. The instructor will be able to see the original data entry and the changes that were made. Also, LabArchives Classroom Edition provides secure and redundant data storage in the cloud through primary and disaster recovery servers, thus archiving the original data permanently. Therefore, by using LabArchives we were able to preserve the originality of data.

Students were required to enter data as it was generated while doing the experiment. Thus, all data entries should be made during the lab period or shortly afterwards. After data entry students submitted their lab notebook pages for review, and the submissions were time stamped. Any data submitted late was considered invalid. Thus, using LabArchives we were able to ensure that data was recorded contemporaneously (or simultaneously) with the experiment. Data generated in the bioprocess engineering lab course had a mix of computer-generated data which were uploaded to LabArchives and non-computer-generated data that were entered manually. Accuracy of computer-generated data was confirmed through a combination of Lab Archives audit trail and time stamp on the computer on which the data was generated. Accuracy of non-computer-generated data was confirmed manually by random spot checks by the TA or the instructor. Thus using an ELN enables superior data integrity as defined in ALCOA with much less effort than is possible through PLNs.

### Assessment of good documentation practices through ELN

The ability to grade lab notebook pages and send feedback to students through LabArchives was helpful in assessing students’ good documentation practices. LabArchives is cloud-based and did not require instructors to remove notebooks from students for grading. Instructors added comments, highlighted mistakes, and explained grading directly in the notebook, which was visible to all team members. Given the limited room available in a PLN, confusion often arises due to illegibility of feedback. Since feedback was communicated to students instantly, they could implement the feedback in their next ELN entries. As students became familiar with ELN their average grade for GDPs improved. A recent study on the use of LabArchives in an upper-level biomedical engineering lab course reported improvement in students’ documentation and communication skills when using ELN compared to PLN [26].

Student competency in GDPs were graded according to the rubric given in Table 2. The rubric was designed to quantify the mastery in attributes important to GDPs and learning. The process of collaborative protocol revision with the instructor/TA was weighted highest because this is the step where students’ conceptual understanding of the experiment is challenged and it presents many teaching opportunities. Raw data entry was weighted next highest. This attribute measured the level of mastery in recording data compliant with ALCOA, including good documentation practices. Draft protocol and the list of objectives measured the students’ level of preparation for the experiment. Therefore, using ELN students were trained in GDPs which is crucial for data reproducibility and reliability [5].

**Table 2:** Rubric for assessing good documentation practices using ELN

Attribute	% contribution to ELN grade	Competencies measured
Draft protocol	16.5	Draft version of complete protocol is submitted before the lab class begins. All obvious steps are covered in the protocol, and the protocol reflects some understanding of theory behind the experiments.
Objectives	16.5	Check if experimental objectives are clearly stated, preferably as a bullet point listing.
Revised Protocol	42	Students actively participated in collaborative revision of their draft protocol with the instructor/TA. Arguments for experimental steps were strong.
Raw data and good documentation	25	Check if all raw data was compliant with ALOCA.

ELN contributed to 20% of the lab course grade

### User experiences and improvements to ELN implementation

**Fig. 4:**
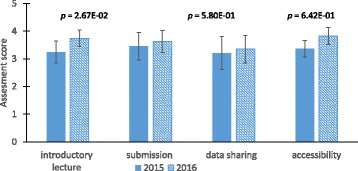
User assessment scores for various attributes from 2015 and 2016 course offerings. Data is represented as mean ± standard deviation. Statistical significance is evaluated using a two sample *t*-Test assuming unequal variances. Data categories include: introductory lecture = LabArchives introductory lecture; submission = turning in protocols and lab notebook pages; data sharing = sharing data with other teams; accessibility = accessibility of information needed for the experiments in LabArchives. *n* = 32 for 2015 and *n* = 23 for 2016

After the bioprocess engineering lab course offerings in 2015 and 2016 students were asked to complete an exit survey on ELN use. Data was extracted using the analytics functionality in SurveyMonkey, and analyzed to determine user experiences. The survey presented students with questions that addressed a range of ELN user experiences during the lab course. Along with quantitative ratings of several attributes, students were also encouraged to explain or comment on their ratings. Comparison of quantitative student responses between 2015 to 2016 show statistical differences in some attributes, while other attributes had little to no change (Fig. 4).

During both offerings of the lab course, an introductory lecture on LabArchives and ELN use was presented to students. In 2015, the introductory lecture consisted of an overview of LabArchives and how it would be implemented throughout the course. In 2016, the introductory lecture was presented along with a supplementary template that included a hands-on demonstration on the use of LabArchives. Quantitative responses pertaining to the introductory lecture and ELN accessibility of information, indicated a statistically significant increase in positive attitudes between 2015 and 2016 (Fig. 4). In contrast, there was no statistical change in student attitudes toward submitting assignments or data sharing through the ELN system.

Students were asked about their experience with the introductory lecture to determine what aspects of the introduction were helpful and what might be changed to improve the lecture. The positive change in attitudes toward the introductory lab lecture can be attributed to the additional supplementary teaching tools that were added into the 2016 presentation. Significant increase in accessibility is also likely due to the introductory lectures’ supplementary additions that offered a visual aid and therefore a stronger orientation for students to navigate LabArchives. Most student ratings on all attributes studied was on average between 3 (neutral) and 4 (very good) suggesting that they were satisfactory experiences, but there is also room for improvement.

**Fig. 5:**
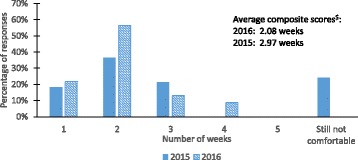
Comparison of time taken for students to become familiar with LabArchives between 2015 and 2016 lab course offerings. ^$^Average composite score of student responses, weighting students who reported “still not comfortable” as 6 weeks. n = 32 for 2015 and n = 23 for 2016

Students reported varied amounts of time that it took for them to become comfortable with the use of LabArchives (Fig. 5). In 2015, 24% of students reported that they were still not comfortable with ELN use at the end of 10-week lab course. In contrast, during the 2016 course offering, all students responding to the survey reported that they were comfortable with ELN use by the fourth week of the course. Further, a majority of students in 2016 reported 2 weeks as the amount of time it took for them to become comfortable with Lab Archives. To quantify the amount of time it took for students to become comfortable with LabArchives, we estimated a weighted average composite score. The average time it took for students to become comfortable with LabArchives decreased from approximately 3 weeks in 2015 to approximately 2 weeks in 2016, indicating a positive trend in student comfort levels. The addition of teaching supplements to the 2016 introductory lecture likely contributed to the reduction in learning period from 3 to 2 weeks.

**Fig. 6:**
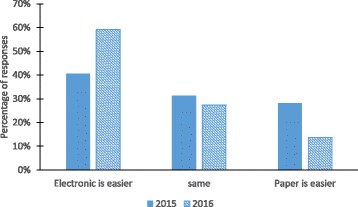
Student preferences of ELN vs PLN for compliance from 2015 to 2016 lab course offerings. n = 32 for 2015 and n = 23 for 2016

**Fig. 7:**
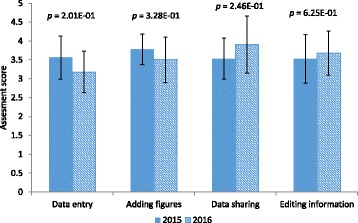
Comparison of student experiences in completing critical documentation tasks using ELN and PLN. Statistical significance is evaluated using a two sample *t*-Test assuming unequal variances. n = 32 for 2015 and n = 23 for 2016

When asked which system makes it easier to comply with academic rules and expectations for the lab course, approximately 40% of students reported a preference for ELN in 2015, while approximately 60% of students chose ELN over PLN in 2016. In 2015, close to 30% of students reported a preference for paper, while that percentage drops to approximately 14% in 2016 (Fig. 6). There was minor difference between offerings in the number of students who reported that PLN and ELN were the same regarding compliance. It should be noted that between 2015 and 2016 course offerings, LabArchives made several updates and enhancements to the software related to improving user experience. The increase in students’ preference for ELN in an academic laboratory setting was a culmination of improvements made by instructors and the LabArchives software. When students were asked to compare their experiences in using ELN vs. PLN to complete tasks critical for good documentation and compliance, most ratings for all tasks were 3 (ELN and PLN are the same) or 4 (ELN is better than PLN) with average ratings ranging from 3.18 to 3.9 (Fig. 7). Students in both course offerings had a better experience in using ELN over PLN for data entry, adding figures, data sharing (for collaboration), and editing data and protocols. The better student experience in using ELN can be related to their higher preference for using ELN over PLN. We continue to improve student experiences in completing these critical tasks through additional coaching and demonstration videos so students can fully utilize the potential of ELNs.

## Conclusions and future directions

We successfully implemented a cloud-based ELN in our bioprocess engineering lab course to: (i) train students in GDPs, (ii) facilitate data sharing and collaboration, (iii) enhance communication between students and instructors, and (iv) achieve superior data integrity. Cloud-based ELNs are no more expensive than PLNs, but do tremendously improve the logistics of experiment workflow. Instructors have greater control of the lab experiments and can provide quick and streamlined feedback to students. Through the robust audit trail in ELNs, the lab course was compliant with all elements of data integrity. ELNs also facilitate easy archiving of data. Historical data is now readily available for future reference, unlike PLNs which are typically discarded after the course and the data is permanently lost. Since space is not an immediate limitation, ELNs can accommodate descriptive comments, protocol revisions, and data revisions as necessary, all of which are archived with a clear audit trail. ELN thus becomes a true historical record of the lab course.

There was generally a favorable response from students in using ELN. Their experiences were more favorable during the second time we offered ELN in 2016. The changes we implemented in ELN instruction and the upgrades to LabArchives software after the 2015 offering led to a better student experience in 2016. We expect the student experience to improve further as we continue to make changes in the future. Since ELN is new to most students, a learning curve of about 2 weeks was observed. After this initial learning period, we did not observe any significant problems in ELN use by students. For the instructor and TAs, ELNs facilitated online, remote grading without the need to handle heavy PLNs. Grades and feedback were immediately available to students unlike PLNs where the students need to wait for graded PLNs to be returned. ELN provided a single platform where instructors and TAs can post documents related to experiments, check experimental data, and monitor data sharing between teams, all remotely if needed. The audit trail in ELN helped instructors to maintain superior data integrity without actively monitoring for breach of data integrity. The instructors and TAs need to get trained on ELN use, and students go through a learning curve, but benefits of ELN outweigh the initial extra effort. Moreover, course data and information in ELN can transferred from previous course offerings, thus making is easy to repeat courses. ELNs can be implemented in classes of all sizes, since uploaded course information can be disseminated to any number of students immediately, and grading and providing feedback is simpler than PLNs. In the future, we plan to integrate a Laboratory Information Management Software (LIMS) with ELN to make this lab course truly paperless. We will use LIMS to manage lab inventory and to track archived samples. As noted by Sayre et al., documentation requirements of every lab will be unique, and the choice of ELN will depend on cost, compatibility, access control, type of data, and the specialized functionalities required [27]. The use of ELN described in this paper worked seamlessly for the bioprocess engineering lab course and can be adapted to other life science and engineering lab courses.

## Abbreviations

ALCOA: Attributable, legible (and long lasting), contemporaneous, original, and accurate
ELN: Electronic lab notebook
FDA: Food and Drug Administration
GDP: Good documentation practice
LIMS: Laboratory Information Management System
NIH: National Institutes of Health
PLN: Paper lab notebook
TA: Teaching assistant
